# Highly efficient and stable inverted perovskite solar cell employing PEDOT:GO composite layer as a hole transport layer

**DOI:** 10.1038/s41598-018-19612-7

**Published:** 2018-01-18

**Authors:** Jae Choul Yu, Ji A Hong, Eui Dae Jung, Da Bin Kim, Soo-Min Baek, Sukbin Lee, Shinuk Cho, Sung Soo Park, Kyoung Jin Choi, Myoung Hoon Song

**Affiliations:** 1 School of Materials Science Engineering and KIST-UNIST Ulsan center for Convergent Materials/Low Dimensional Carbon Center/Perovtronics Research Center, Ulsan National Institute of Science and Technology (UNIST), UNIST-gil 50, Ulsan, 44919 Republic of Korea; 2 School of Materials Science and Engineering, Ulsan National Institute of Science and Technology (UNIST), UNIST-gil 50, Ulsan, 44919 Republic of Korea; 3 Department of Physics and EHSRC, University of Ulsan, Ulsan, 44610 Republic of Korea

## Abstract

The beneficial use of a hole transport layer (HTL) as a substitution for poly(3,4-ethlyenedioxythiophene): polystyrene sulfonate (PEDOT:PSS) is regarded as one of the most important approaches for improving the stability and efficiency of inverted perovskite solar cells. Here, we demonstrate highly efficient and stable inverted perovskite solar cells by applying a GO-doped PEDOT:PSS (PEDOT:GO) film as an HTL. The high performance of this solar cell stems from the excellent optical and electrical properties of the PEDOT:GO film, including a higher electrical conductivity, a higher work function related to the reduced contact barrier between the perovskite layer and the PEDOT:GO layer, enhanced crystallinity of the perovskite crystal, and suppressed leakage current. Moreover, the device with the PEDOT:GO layer showed excellent long-term stability in ambient air conditions. Thus, the enhancement in the efficiency and the excellent stability of inverted perovskite solar cells are promising for the eventual commercialization of perovskite optoelectronic devices.

## Introduction

Organic-inorganic perovskite structures have been regarded as promising next-generation optoelectronic materials due to their superior optical and electrical properties, simple solution processing, and low cost^1–5^. In particular, the power conversion efficiency (PCE) values of perovskite solar cells have increased over the past few years from 3.8% to more than 22%^6–16^. Moreover, the external quantum efficiency (EQE) values of the perovskite light-emitting diodes (PeLEDs) also have increased to 9.3% for green emission and 11.7% for near-infrared emission^17–23^.

The structure of perovskite devices is classified into conventional and inverted categories. Although conventional perovskite solar cells generally operate at high efficiencies, they are not suitable for flexible devices because they contain a metal oxide as an electron transport layer, which is rigid and requires high-temperature processing for good electrical properties^12,13,16^. On the other hand, inverted perovskite solar cells consisting of ITO/poly(3,4-ethlyenedioxythiophene):polystyrene sulfonate (PEDOT:PSS)/perovskite/PCBM/Ag where ITO is indium-tin-oxide and PCBM is the fullerene derivative phenyl-C61-butyric acid methyl ester, can be fabricated by simple, low-temperature (150 °C) processing methods^24–27^. PEDOT:PSS is widely used as a hole transport layer (HTL) in organic and perovskite optoelectronic devices because it provides a smooth surface on the ITO electrode. However, the high acidity of the PEDOT:PSS solution corrodes the ITO electrode and perovskite, which limits the stability and performance of organic and perovskite optoelectronic devices^28,29^. Moreover, the inverted perovskite solar cells based on PEDOT:PSS exhibit low open circuit voltage (*V*_oc_) and short circuit current (*J*_sc_). This electrical disadvantage is due to the larger energy barrier between the perovskite and PEDOT:PSS layers, which limits charge extraction, and the poor electron blocking ability necessary for suppressing leakage current^30^. Therefore, researchers are investigating a variety of materials as replacements for PEDOT:PSS, including metal oxides and doped or solvent-treated PEDOT:PSS layers^31–34^.

The metal oxides, such as nickel oxide (NiO_x_) and Cu-doped nickel oxide (Cu:NiO_x_), significantly enhance carrier mobility and air stability compared to PEDOT:PSS, resulting in the improvement of the device performance and long-term stability^31,35,36^. Doped PEDOT:PSS exhibits better electrical conductivity and a lower highest occupied molecular orbital (HOMO) energy level, resulting in improvement of *J*_sc_, *V*_oc_ and overall device performance^32^. Recently, Chen *et al*. have reported that the use of the solvent Dimethylformamide (DMF) to treat PEDOT:PSS improves device performance by enhancing electrical conductivity of PEDOT:PSS and reducing surface roughness^33^. Unfortunately, these doped and solvent-treated PEDOT:PSS methods do not improve the device stability, which is why the stability still needs to be systemically studied.

In this work, we demonstrated highly efficient and stable perovskite solar cells by replacing the PEDOT:PSS with a PEDOT:PSS and a graphene oxide (PEDOT:GO) composite layer that acts as an HTL. The PEDOT:GO composite film exhibited a higher work function and better electrical conductivity compared to PEDOT:PSS. Moreover, the PEDOT:GO composite layer enhanced the crystallinity of the perovskite film and remarkably reduced leakage current in the perovskite solar cell. The optimized PEDOT:GO-based perovskite solar cell showed a significantly enhanced PCE of 18.09% with negligible *J*-*V* hysteresis. In addition, the encapsulated perovskite solar cell with the PEDOT:GO composite layer showed excellent long-term stability under ambient operating conditions.

## Results

**Figure 1:**
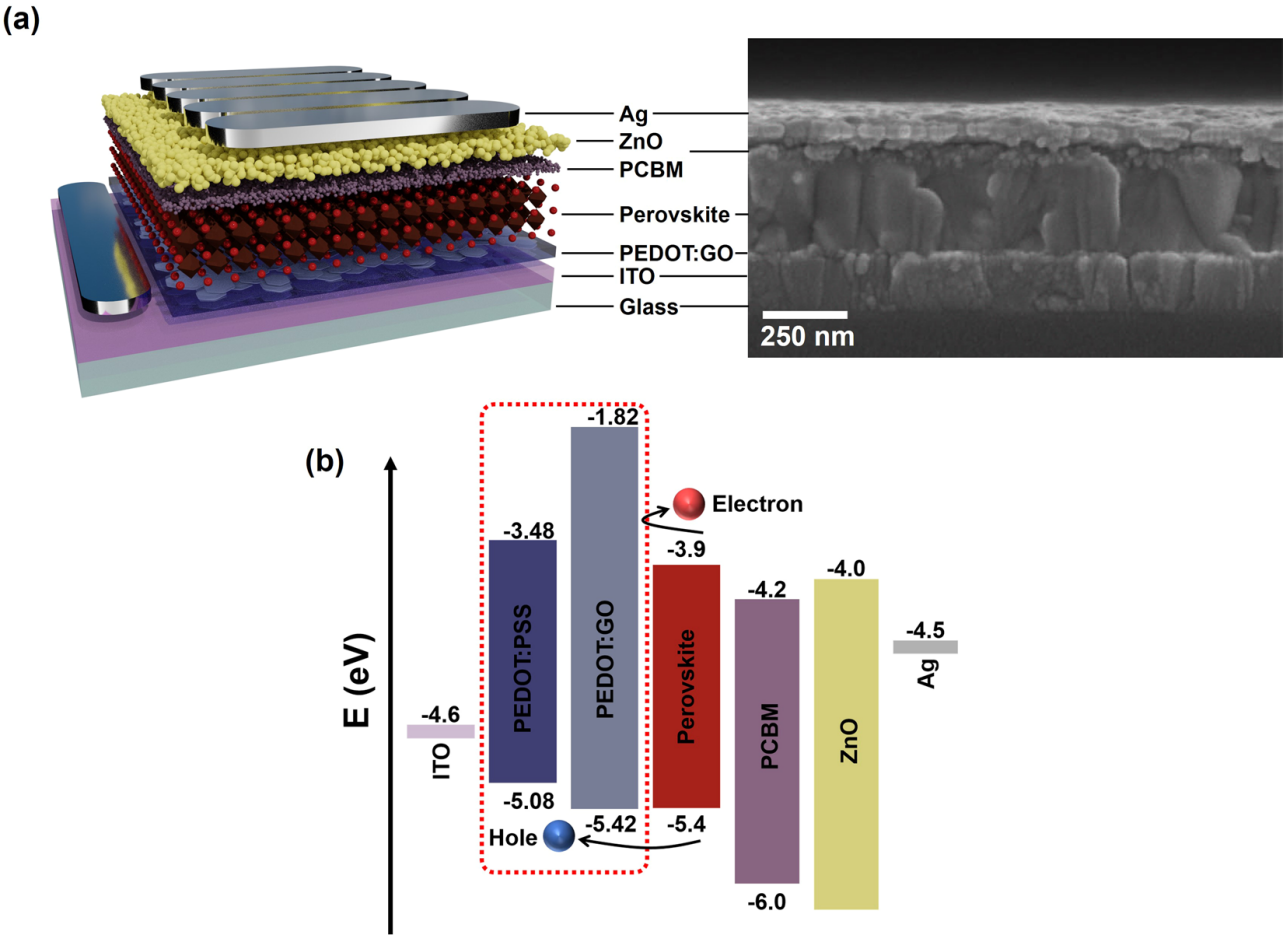
(**a**) The device schematic and SEM cross-sectional image of a perovskite solar cell (ITO/HTL/perovskite/PCBM/ZnO nanoparticles/Ag) and (**b**) relative energy levels of the various device components in the perovskite solar cells.

Figure 1(a) and (b) show the corresponding device schematic and cross-sectional scanning electron microscope (SEM) image and relative energy levels of components in the whole device consisting of ITO/HTL/perovskite/PCBM/ZnO nanoparticles/Ag, respectively. To understand how PEDOT:GO-based perovskite solar cells may improve efficiency and stability compared to PEDOT:PSS, we observed the structure and morphology of precursors and the final composite. The GO film prepared with pristine GO solution (Highly Concentrated Graphene Oxide, Graphene Supermarket) exhibited a very large and non-uniform GO flake size of about 10 µm, as shown in Fig. S1(a). To obtain a small and uniform size of GO flakes, the pristine GO solution was ultrasonicated and the *p*H value was adjusted to *p*H 9. After 5 hours of sonication, a homogeneous dispersion of 500 nm sized GO flakes was obtained, as shown in Fig. S1(c). The sizes of GO flakes change with time of sonication. SEM images of the PEDOT:GO composite film fabricated with these homogeneous GO flakes (5 hours of sonication) showed a similar morphology to pristine PEDOT:PSS films, whereas the PEDOT:GO composite film with non-uniform and large GO flakes (without and with sonication for 0.5 h) showed a significantly rougher surface, as shown in Fig. S2.

To further analyze the morphology of perovskite film further, the perovskite film prepared on pristine PEDOT:PSS and PEDOT:GO composite films (with various flake sizes of GO) were observed using atomic force microscopy (AFM), as shown in Fig. S3. The perovskite films deposited onto PEDOT:GO composite layers (where GO was sonicated for 5 h) and pristine PEDOT:PSS showed a similar morphology, with low root-mean-square (RMS) roughness values of 16.0 and 15.2 nm, respectively. However, the perovskite films prepared on PEDOT:GO composite layers (without and with sonication for 0.5 h) exhibited a significantly rougher surface with high RMS roughness values of 23.4 and 18.8 nm, respectively.

**Figure 2:**
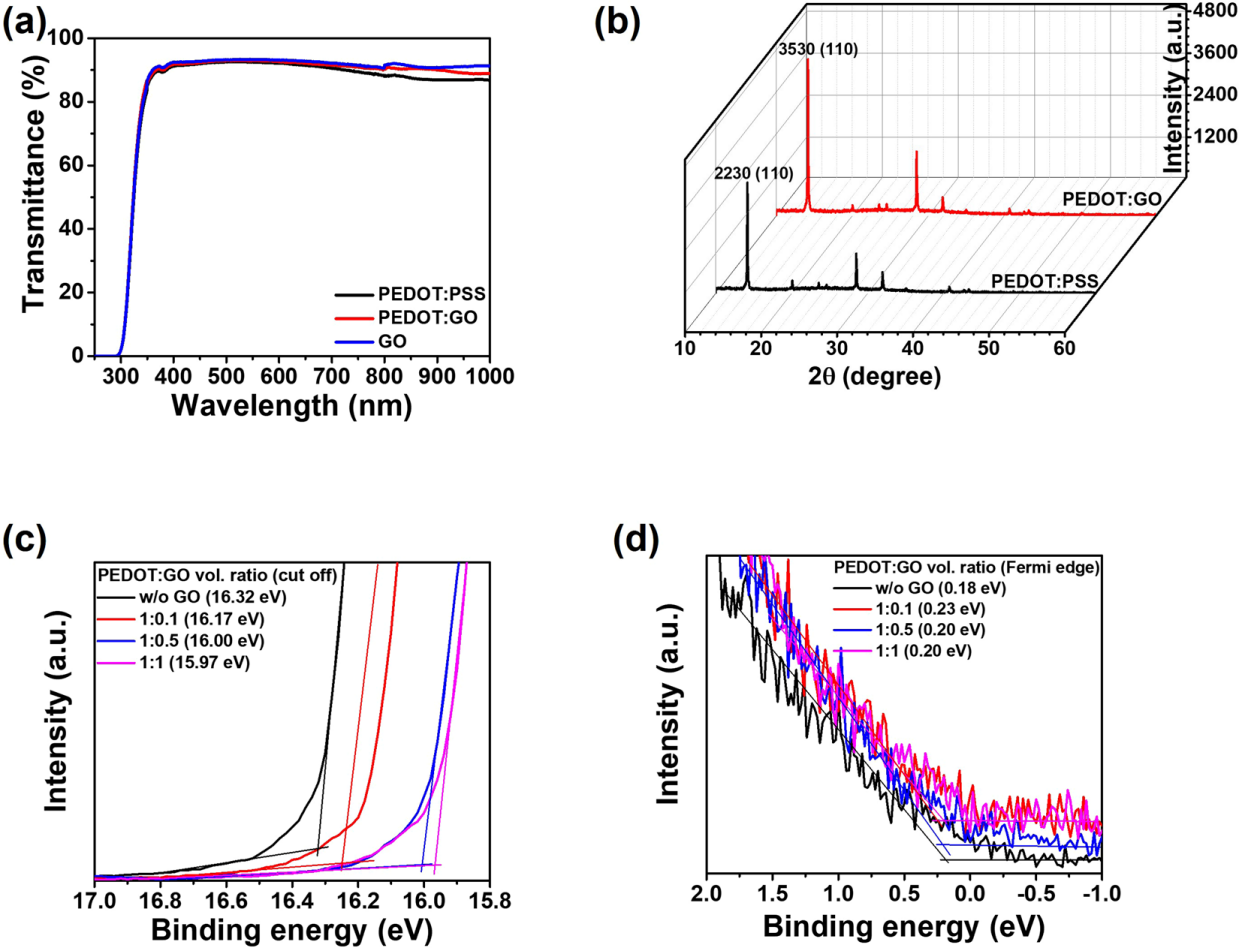
(**a**) Transmittance spectra of PEDOT:PSS, PEDOT:GO composite, and GO, (**b**) XRD patterns of perovskite prepared on PEDOT:PSS and PEDOT:GO composite films with preferred orientation along the (110) directions. The UPS measurements of PEDOT:PSS and PEDOT:GO composite (vol. ratio of 1:0.1, 1:0.5, and 1:1) films; (**c**) secondary electron cutoff regions and (**d**) Fermi edge (valence band edge) regions, respectively.

To investigate the effect of a PEDOT:GO composite layer on perovskite films and devices, the transmittance of the PEDOT:GO composite film was measured and correlated to the crystallinity. Since sunlight reaches the perovskite layer after passing through its HTL, high optical transparency of an HTL is necessary for efficient light harvesting and improved absorbance of the perovskite film. To confirm the transparency of HTLs, the transmittance spectra of PEDOT:PSS, PEDOT:GO composite (vol. ratio of 1:0.5), and GO films all with film thicknesses of 30 nm were measured, as shown in Fig. 2(a). The GO film alone had the highest transparency above 700 nm, and the PEDOT:GO composite film showed higher transmittance above 700 nm than the pristine PEDOT:PSS film, attributed to the higher transparency of the GO film. In particular, the transmittance of the PEDOT:GO composite film between 800 and 1000 nm was excellent, which would be significant if used as an HTL for Si/perovskite tandem solar cells.

The crystallinity of the perovskite film prepared on pristine PEDOT:PSS and PEDOT:GO composite layers was confirmed by X-ray diffraction (XRD) spectra in Fig. 2(b). The peak at 14.10° corresponds to the (110) orientation of the perovskite film, and the peak intensity of that orientation for the PEDOT:GO composite (vol. ratio of 1:0.5) was 160% of that for the pristine PEDOT:PSS layer. It has been previously reported that the crystallinity of perovskite films prepared on a GO layer increases because perovskite crystals preferentially grow on the GO layer rather than the PEDOT:PSS film^37^. Therefore, the enhancement of the crystallinity of the perovskite layer may originate from the presence of GO in the PEDOT:GO composite film. Structural changes that occur as a result of improved perovskite crystallinity on the PEDOT:GO composite film may also influence the device efficiency. The preferred orientation of (110) in perovskite films has been reported to increase the fill factor (*FF*) and *V*_oc_ in perovskite solar cells^38^.

In addition, the work functions of the PEDOT:PSS and PEDOT:GO composites (vol. ratio of 1:0.1, 1:0.5 and 1:1) were measured using ultra-violet photoemission spectroscopy (UPS) to investigate the change of the work function due to the presence of GO in PEDOT:PSS. Figure 2(c) and (d) show the binding energy at secondary electron cut-off and Fermi-edge regions, respectively. The work functions of the PEDOT:PSS, PEDOT:GO composities (vol. ratio of 1:0.1, 1:0.5 and 1:1) were determined to be 4.90, 5.05, 5.22 and 5.25 eV, respectively. The highest occupied molecular orbital (HOMO) levels are calculated by the sum of the work functions and Fermi edge enegy^39^. Therefore, the values of HOMO levels for the PEDOT:PSS and PEDOT:GO composite (vol. ratio of 1:0.1, 1:0.5 and 1:1) were determined to be 5.08, 5.28, 5.42 and 5.45 eV. Interestingly, the work function of PEDOT:GO increased from 4.90 eV to 5.25 eV depending on the vol. ratio of GO in PEDOT:PSS. In fact, the benzoid-quinoid transition of PEDOT structure after mixing the GO and PEDOT:PSS solution was observed because both the highly oxidized functional group and the sp^2^ conjugation of GO bring about hydrogen bonding and π-π stacking in the previous study^40^. A few reports have demonstrated that the work function of PEDOT:PSS increases when the structure of PEDOT changes from benzoid to quinoid^41,42^. Therefore, the increase of work function of PEDOT:GO may originate from the benzoid-quinoid transition of PEDOT.

To confirm the correlation between improved transmittance, crystallinity, and efficiency of the perovskite on the PEDOT:GO composite film and performance of a device made with this composite, perovskite solar cells prepared on pristine PEDOT:PSS and PEDOT:GO composite films were fabricated. Unfortunately, the PEDOT:GO composite-based devices produced with the large GO particles showed lower power conversion efficiencies (PCEs) than the reference cell, as shown in Fig. S4. It is well-known that device performance deteriorates in perovskite-based optoelectronic devices when the morphology of the perovskite film is deficient^9,43,44^. However, the PEDOT:GO composite device fabricated with fine, uniform GO particles (where GO was sonicated for 5 h) exhibited a PCE of 16.16%, higher than the reference cell at 13.59%. The detailed photovoltaic parameters are summarized in Table S1.

Particle size and uniformity of GO affected the PCE, and the concentration of GO in PEDOT:GO composite layer also affected device performance, as confirmed in Fig. S5. The *J*-*V* curves of perovskite solar cells with various volume ratios of GO in PEDOT:GO composite layers (with GO particle sonication for 5 h) were determined. The device with PEDOT:GO composite layer (vol. ratio of 1:0.5) exhibited the highest PCE of 17.74%, with a higher *J*_sc_, *FF*, and *V*oc than other GO concentrations tested. However, an excessive amount of GO impaired device performance because the insulator property of GO reduces the charge extraction characteristics. The detailed photovoltaic parameters are summarized in Table S2.

**Figure 3:**
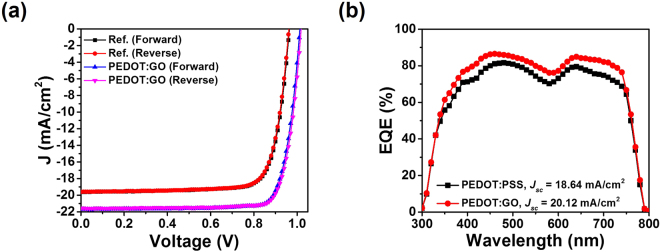
(**a**) *J*-*V* curves of the best-performing perovskite solar cells with PEDOT:PSS and a PEDOT:GO composite film under forward and reverse bias. (**b**) The external quantum efficiency (EQE) spectra of the best-performing cells.

The parameters of *J*_sc_, *FF, V*_oc_, and PCE were also measured to determine the quality of the device performance. Figure 3(a) and (b) show the *J*-*V* curves and EQE spectra of the best performing devices with PEDOT:GO and pristine PEDOT:PSS film as HTLs, respectively. The best performance of a perovskite solar cell with a PEDOT:GO composite layer corresponded to a *J*_sc_ of 21.55 mA/cm^2^, an *FF* of 82.3%, a *V*_oc_ of 1.02 V, and a PCE of 18.09%. Weaker results were found for the best performing perovskite solar cell with a PEDOT:PSS film, where we found a *J*_sc_ of 19.61 mA/cm^2^, an *FF* of 78.53%, a *V*_oc_ of 0.97 V, and a PCE of 14.95%, as shown in Fig. 3(a). A summary of the performance parameters for the best-performing solar cells is presented in Table 1.The perovskite solar cell with the optimized PEDOT:GO composite film exhibited higher EQE values in the overall range compared to the reference device, which supports the notion that the device with a PEDOT:GO composite layer facilitated efficient charge extraction and higher *V*oc. In addition, both devices showed negligible *J*-*V* hysteresis. The steady-state photocurrent and output efficiency (SPO) was measured at the 0.87 V, as shown in Fig. S6. The perovskite solar cell with PEDOT:GO composite film achieved a stabilized current density of 20.30 mAcm^−2^ and a stabilized PCE of 17.68%. This result supports the negligible *J*-*V* hysteresis of the perovskite solar cell with PEDOT:GO composite film.

**Table 1:** Summary of the device performance of perovskite solar cells with PEDOT:PSS and PEDOT:GO composite film under forward and reverse bias.

Devices configuration	J SC [mA/cm]	V OC [V]	FF [%]	η[%]
ITO/PEDOT:PSS/perovskite/PCBM/ZnO NPs/Ag (Forward)	19.61	0.97	78.41	14.91
ITO/PEDOT:PSS/perovskite/PCBM/ZnO NPs/Ag (Reverse)	19.63	0.97	78.53	14.95
ITO/PEDOT:GO/perovskite/PCBM/ZnO NPs/Ag (Forward)	21.55	1.02	81.2	17.96
ITO/PEDOT:GO/perovskite/PCBM/ZnO NPs/Ag (Reverse)	21.55	1.02	82.3	18.09

**Figure 4:**
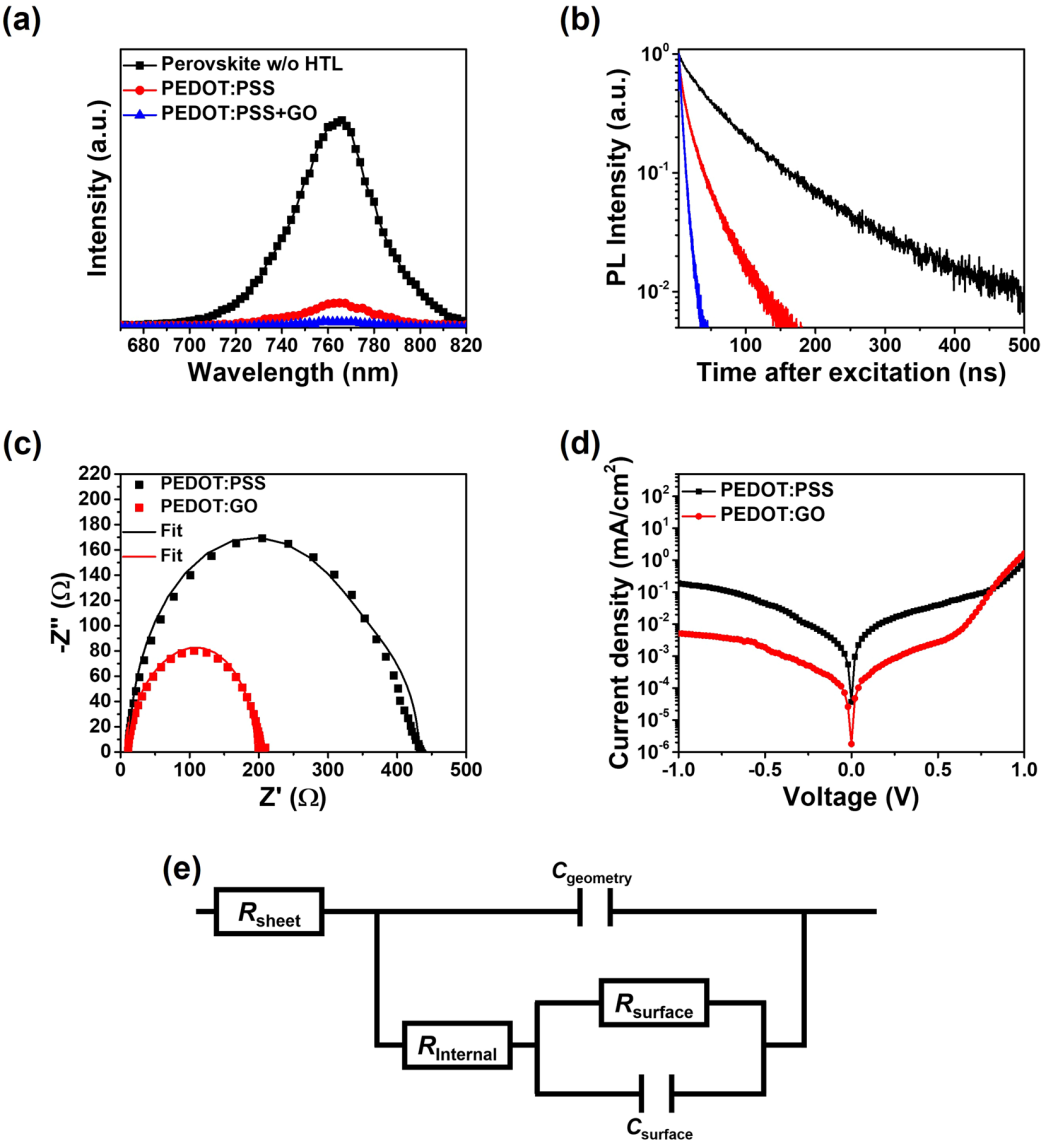
(**a**) Steady-state PL spectra and (**b**) time-resolved PL spectra of perovskite films prepared on PEDOT:PSS and PEDOT:GO composites, respectively. (**c**) Nyquist plots of perovskite solar cells with PEDOT:PSS and PEDOT:GO composite films. (**d**) Logarithmic plot of *J*-*V* characteristics of perovskite solar cells measured under the dark. (**e**) The equivalent circuit model for solar cells.

To further investigate the effect of a PEDOT:GO composite film on the performance of the perovskite solar cell, the charge carrier dynamics via a time-correlated single photon count (TCSPC) and electrochemical impedance spectroscopy (EIS) were measured. Figure 4(a) and (b) show steady-state and time-resolved photoluminescence (PL) spectra of perovskite layers prepared on a PEDOT:PSS film and a PEDOT:GO composite film (vol. ratio of 1:0.5), respectively. The PL intensity of the perovskite film prepared on the PEDOT:GO composite decreased about 97%, whereas the PL intensity of perovskite film prepared on PEDOT:PSS film decreased about 88%. Moreover, the exciton lifetime of the perovskite film prepared on a PEDOT:GO composite exhibited a remarkable decrease (3.48 ns) compared to the perovskite film prepared on PEDOT:PSS film (14.76 ns). Consequently, the PL intensity and exciton lifetime of the perovskite film prepared on PEDOT:GO composite film showed a significant decrease compared to perovskite films without an HTL (PEDOT:PSS) because of a quenching effect. Thus, these results support improved hole extraction toward the anode using a PEDOT:GO composite layer as a HTL in the perovskite solar cells^32^. The corresponding parameters describing exciton lifetimes are also summarized in Table S3.

We also conducted electrochemical impedance spectroscopy (EIS) measurements of our perovskite solar cells to investigate the inner series resistances, which consist of sheet resistance (*R*_sheet_) of the electrodes, internal resistance (*R*_internal_) of device and surface recombination resistance (*R*_surface_) at the interface between the adjacent charge carrier layer and electrode. Figure 4(c) shows the Nyquist plots of the perovskite solar cells with the PEDOT:GO composite and the PEDOT:PSS films. The equivalent circuit used for these devices is shown in Fig. 4(e). The experiment data were well described by the equivalent circuit and the fitting parameters, as shown in Fig. 4(c) and Table S4, respectively. In particular, the *R*_internal_ values exhibited 357.8 and 133.7 Ω for perovskite solar cells with PEDOT:PSS and PEDOT:GO composite films as HTLs, respectively. The smaller *R*_internal_ of the perovskite solar cell with PEDOT:GO composite layer is attributed to an enhanced charge extraction through energy level matching between HTL and perovskite layer and the enhancement of conductivity of PEDOT:GO, resulting in improved device photocurrent as well as improved PCE. Moreover, the suppression of leakage current was confirmed by the *J*-*V* curves of perovskite solar cells under a dark condition, as shown in Fig. 4(d). The current density of the perovskite solar cell with the PEDOT:GO composite film under reverse bias was approximately two orders of magnitude lower than that of the device with a pristine PEDOT:PSS film. In addition, the different shape of *J*-*V* curves of perovskite solar cells with PEDOT:PSS and PEDOT:GO composite layer occurs at a positive bias because the charge injection of device with PEODT:GO composite layer is more efficient than that with PEDOT:PSS by reducing the injection barrier between a HTL and perovskite layer. This result also supports the notion that the photo-generated charge carriers are effectively extracted through the device^33^.

To investigate the improvement of hole extraction between the HTL and perovskite layer, the current densities of the hole-only devices (ITO/PEDOT:PSS and PEDOT:GO /perovskite/PCBM/ZnO/MoO_3_/Au) were compared, as shown in Fig. S7. The current density of the hole-only device with PEDOT:GO composite film was much larger than that of the hole-only device with pristine PEDOT:PSS film. This result supports the conclusion that the hole extraction in the perovskite solar cell with PEDOT:GO composite film was enhanced due to the higher electrical conductivity of the PEDOT:GO composite film and smaller contact barrier between perovskite layer and the PEDOT:GO. The HTL prepared using the mixture of PEDOT:PSS and graphene oxide (GO) solution for a PEDOT:GO composite layer showed higher electrical conductivity than a PEDOT:PSS film due to the benzoid-quinoid transition, as shown in Table S5, which had also been confirmed in our previous study^40^. Moreover, GO can block the electrons from the perovskite layer because of large band gap, which facilitates the suppression of leakage current, thereby resulting in enhanced device performance.

**Figure 5:**
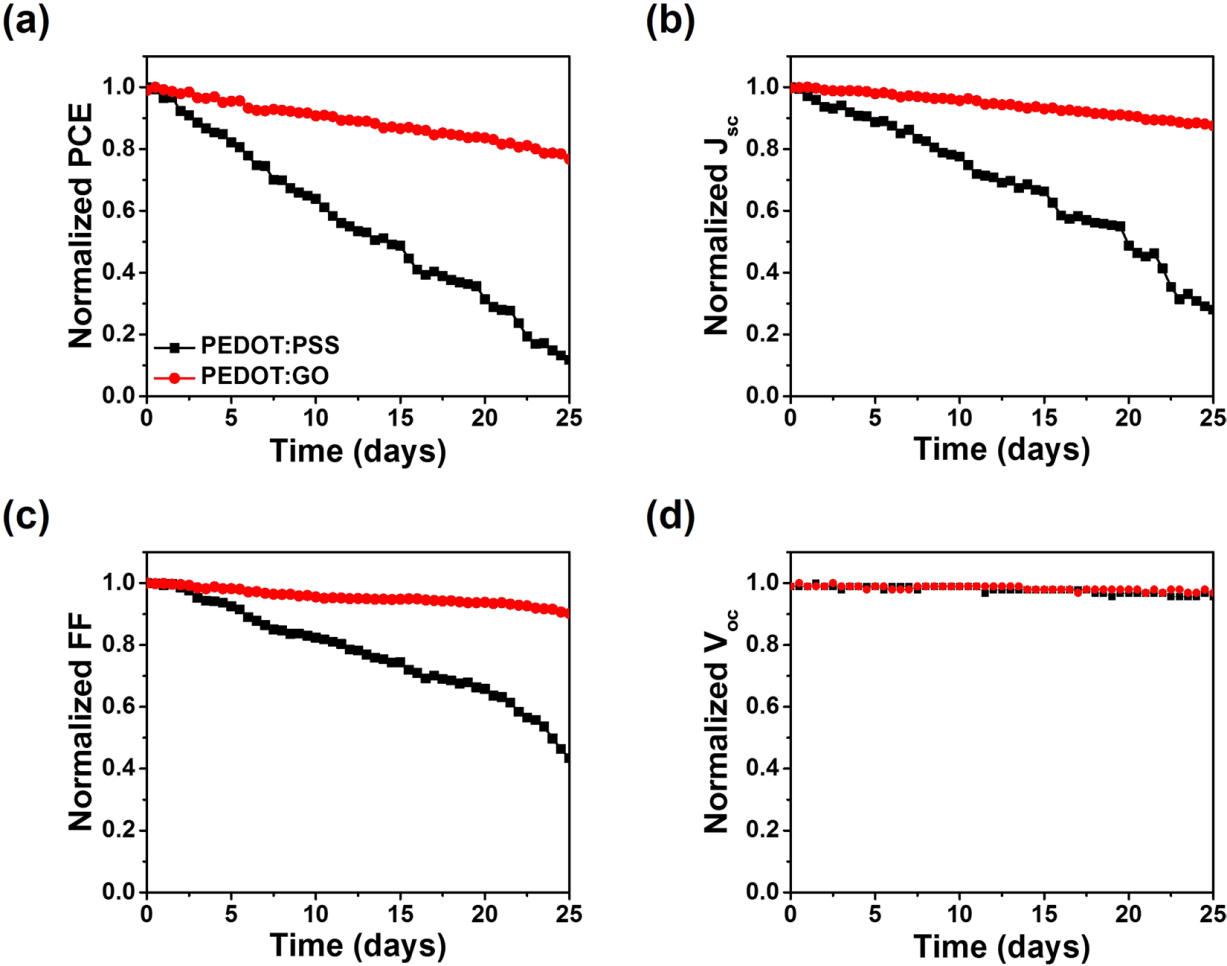
Stability characteristics of the perovskite solar cells with PEDOT:PSS and PEDOT:GO composite films under ambient air conditions: (**a**) Normalized PCE, (**b**) Normalized *J*_sc_, (**c**) Normalized *FF*, and (**d**) Normalized *V*_oc_, respectively.

To confirm the long-term stability of perovskite solar cells, encapsulated devices with PEDOT:PSS and PEDOT:GO composite films were measured under an ambient air condition, as shown in Fig. 5. The PCE of the perovskite solar cell with the PEDOT:PSS film rapidly decreased to 10% of its initial value after 25 days, whereas the perovskite solar cell with the PEDOT:GO composite film maintained 80% of its initial PCE with a much slower degradation rate. The main reason for the PCE deterioration in PEDOT:PSS-based perovskite solar cells stems from the significant decrease of *J*_sc_ and *FF*, which is related to the degradation of ITO and the perovskite layer by highly acidic PEDOT:PSS. This phenomenon is similar to previous reports in the organic photovoltaic field^45,46^. Thus, perovskite solar cell fabricated with a PEDOT:GO composite film was more stable than the reference cell because it reduced the degradation of ITO and perovskite layer by the low acidic property of the GO solution (*p*H 9). It has also been reported that avoiding acidic conditions when processing PEDOT:PSS or GO improves the long-term stability of perovskite solar cells^47,48^.

To increase the statistical significance of our efficiency data, 50 samples of each PEDOT:PSS and PEDOT:GO composite film were fabricated and measured; histograms of *J*_sc_, *V*_oc_, *FF*, and *PCE* for both composites are presented in Fig. S8. The perovskite solar cells with PEDOT:GO composite films exhibited 17.6% higher PCE values and higher values of *J*_sc_, *V*_oc_, and *FF* compared with those with PEDOT:PSS film.

In addition, we fabricated perovskite light-emitting diodes (PeLEDs) to investigate the applicability of a PEDOT:GO composite layer in optoelectronic devices. Figure S9 shows characteristics of PeLEDs with PEDOT:PSS and optimized PEDOT:GO composite films, including the *J*-*V* curve, luminance vs. voltage (*L*-*V*), luminous efficiency vs. voltage, and *EQE* vs. voltage. The PeLED with the PEDOT:PSS film exhibited a maximum luminous efficiency of 3.14 cd/A and an EQE of 0.67%. On the other hand, the PeLED with an optimized PEDOT:GO composite layer exhibited a significantly enhanced maximum luminous efficiency of 10.70 cd/A and a EQE of 2.29%, which represents a significant enhancement of 340%. This enhanced efficiency originates from the improved electrical conductivity and the reduced contact barrier between the PEDOT:GO composite and perovskite films. This result clearly supports the conclusion that the use of a PEDOT:GO composite layer improves the efficiency of perovskite optoelectronic devices. The detailed device performance values of PeLEDs with PEDOT:PSS and PEDOT:GO composite films are summarized in Table S6.

## Discussion

In summary, we demonstrated a simple method for obtaining highly efficient and stable inverted perovskite solar cells by introducing a PEDOT:GO composite film. The inverted perovskite solar cell with an optimized PEDOT:GO composite film showed a PCE of 18.09%, which is higher than that of the perovskite solar cell with a pristine PEDOT:PSS film (14.95%), and both devices had negligible *J*-*V* hysteresis. The high PCE of the perovskite solar cell originated from the superior properties of the PEDOT:GO composite film, including higher optical transmittance over 700 nm, higher electrical conductivity, higher work function to reduce the contact barrier between the perovskite layer and the PEDOT:GO layer, enhanced crystallinity of the perovskite crystal, and suppressed leakage current. These results improved charge carrier extraction and open circuit voltage in the device. In particular, the perovskite solar cell with the PEDOT:GO composite film produced highly enhanced long-term stability with 80% maintenance from its initial PCE after 25 days, while the perovskite solar cell with PEDOT:PSS film showed a decline in PCE. Therefore, we believe that this simple method for introducing PEDOT:GO composite film in perovskite solar cells improves efficiency and long-term stability and facilitates the commercialization of perovskite optoelectronic devices.

## Methods

### Materials

[6,6]-Phenyl-C71-butyric acid methyl ester (PCBM) was purchased from 1-Material. ZnO nanoparticle dispersed in IPA solution was purchased from Nanograde AG (Product No. N-10). The graphene oxide (GO) solution with concentration of 5 g/L in water was purchased from graphene supermarket (Product No. SKU-HCGO-W-175). All other materials were purchased according to the previous report^40^.

### Preparation of the PEDOT:GO composite solution

The GO solution was sonicated for various time (0~5 hrs), and then the PEDOT:GO solution was prepared according to the previous report^40^.

### Device fabrication

The configuration of perovskite solar cells was ITO/HTLs/perovskite/PCBM/ZnO nanoparticle/Ag. The HTLs such as PEDOT:PSS or PEDOT:GO composite were spin-coated onto the pre-patterned ITO substrate at 5,000 rpm for 45 s and annealed at 145 °C for 10 min. The substrates were then transferred into a glove box filled with pure N_2_. Then perovskite precursor solution (1.4 M PbI_2_, 1.33 M MAI and 0.07 M MABr in co-solvent of DMSO:GBL at vol ratio of 3:7) was spin-coated onto PEDOT:PSS and PEDOT:GO composite films via a consecutive 2-step method at 500 rpm and 5000 rpm for 5 and 25 s, respectively. During the spin-casting at 5,000 rpm, the chlorobenzene (300 µL) was dropped onto the substrate and then annealed at 100 °C for 20 min. The PCBM solution dispersed in chlorobenzene (1.77 wt. %) and ZnO nanoparticle dispersion were sequentlly spin-coated onto the perovskite layer at 2,000 rpm and 4,000 rpm for 45 s, respectively. The devices were completely fabricated by thermal deposition of 100 nm silver as a cathode under vacuum condition of <2 × 10^−6^ Torr. The devices area was 0.135 cm^2^ defined by shadow mask.

### Characterization of perovskite film and HTLs

The UPS spectra were measured using an ultra-high vacuum UPS system (ESCALAB 250Xi, Thermo Scientific) at 1 × 10^−9^ torr with a He (21.2 eV) ultraviolet source: all samples were biased at −5V and were measured step size of 0.02 eV. The work function of HTLs (PEDOT:PSS, GO and PEDOT:GO) was calculated using the equation,

1 $$\Phi ={P}_{IN}-({E}_{CE}-{E}_{F})$$

where where *Φ* is the work function, *P*_*IN*_ is the incident photon energy (21.2 eV), *E*_CE_ is the binding energy at cut-off region and *E*_F_ is the binding energy at the Fermi level, which is zero, respectively. The HOMO level are calculated by the sum of the work functions and the Fermi edge energy^39^.

To measure the conductivity of HTLs, 500 nm-thick PEDOT:GO and PEDOT:PSS films were prepared to obtain reliable conductivity values within limited resolution. The electric conductivity of PEDOT:GO and PEDOT:PSS films could be obtained by using 4-point probe measurement (CMT-SR2000N, Korea, measurement range: 1 mΩ/sq~2 MΩ/sq) and thickness measurements with a profilometer (KLA_Tencor, USA). Transmittance of PEDOT:PSS, PEDOT:GO and GO film were evaluated by UV-Vis-NIR spectrophotometer (Agilent Tec., Cary 5000). The impedance spectroscopy was performed using a potentiostat (Gamry Instruments, Reference 600^TM^) at 0.7 V. The oscillating voltage was 10 mV and the frequencies were ranged from 1 MHz to 1 Hz.

## Electronic supplementary material

Supplementary Information
