# Supplementary material for: Highly efficient and stable inverted perovskite solar cell employing PEDOT:GO composite layer as a hole transport layer
Source: Sci Rep. 2018 Jan 18;8:1070. doi: 10.1038/s41598-018-19612-7 (PMC5773582; doi:10.1038/s41598-018-19612-7)
Supplement: Supplementary file 1 — Supplementary Information [file 41598_2018_19612_MOESM1_ESM.doc]

Supplementary Information

**Highly efficiency and stable inverted perovskite solar cell employing PEDOT:GO composite layer as a hole transport layer**

Jae Choul Yu,^1^ Ji A Hong,^1^ Eui Dae Jung,^1^ Da Bin Kim,^1^ Soo-Min Baek,^2^ Sukbin Lee,^1^ Shinuk Cho,^3^ Sung Soo Park,^2^ Kyoung Jin Choi^1^ and Myoung Hoon Song^1*^

*^1^* *School of Materials Science Engineering and KIST-UNIST Ulsan center for Convergent Materials/Low Dimensional Carbon Center/Perovtronics Research Center, Ulsan National Institute of Science and Technology (UNIST), UNIST-gil 50, Ulsan, 44919, Republic of Korea*

*^2^* *School of Materials Science and Engineering, Ulsan National Institute of Science and Technology (UNIST), UNIST-gil 50, Ulsan, 44919, Republic of Korea*

*^3^* *Department of Physics and EHSRC, University of Ulsan, Ulsan, 44610, Republic of Korea*

*To whom correspondence should be addressed. E-mail: [mhsong@unist.ac.kr](mailto:mhsong@unist.ac.kr)

*
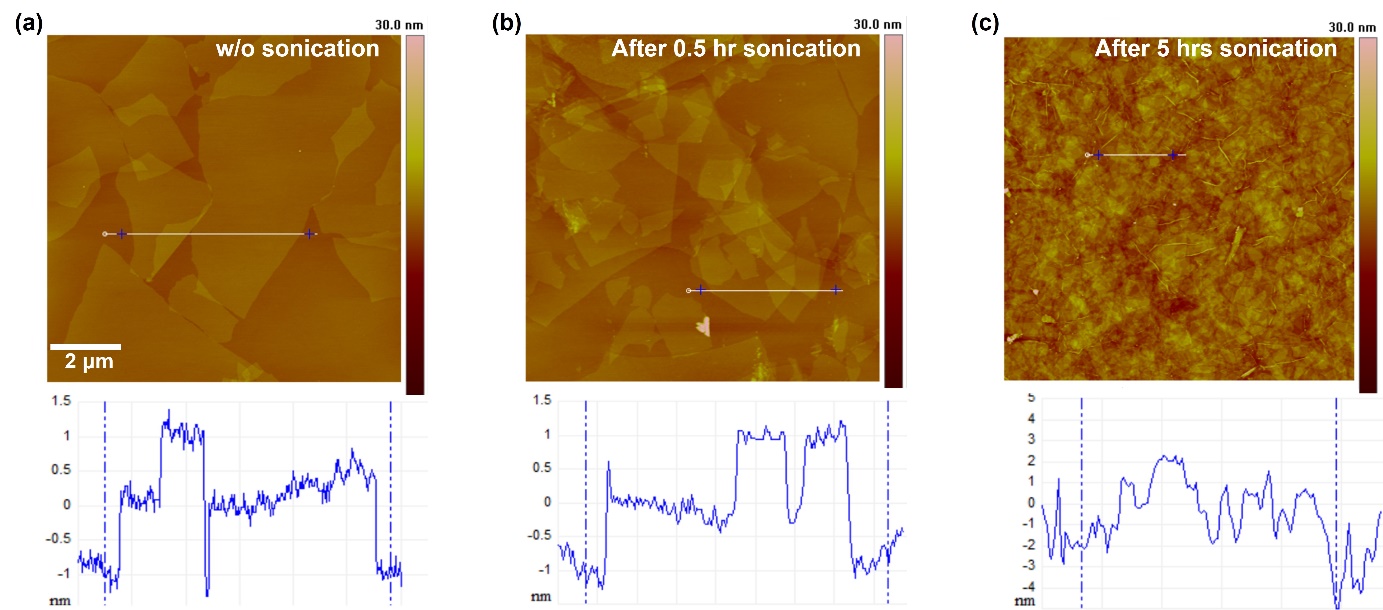
*

**Figure S1.** AFM images of GO films with height profile prepared (a) without sonication (b) with 0.5 h sonication, and (c) after 5 h of sonication, respectively.

**
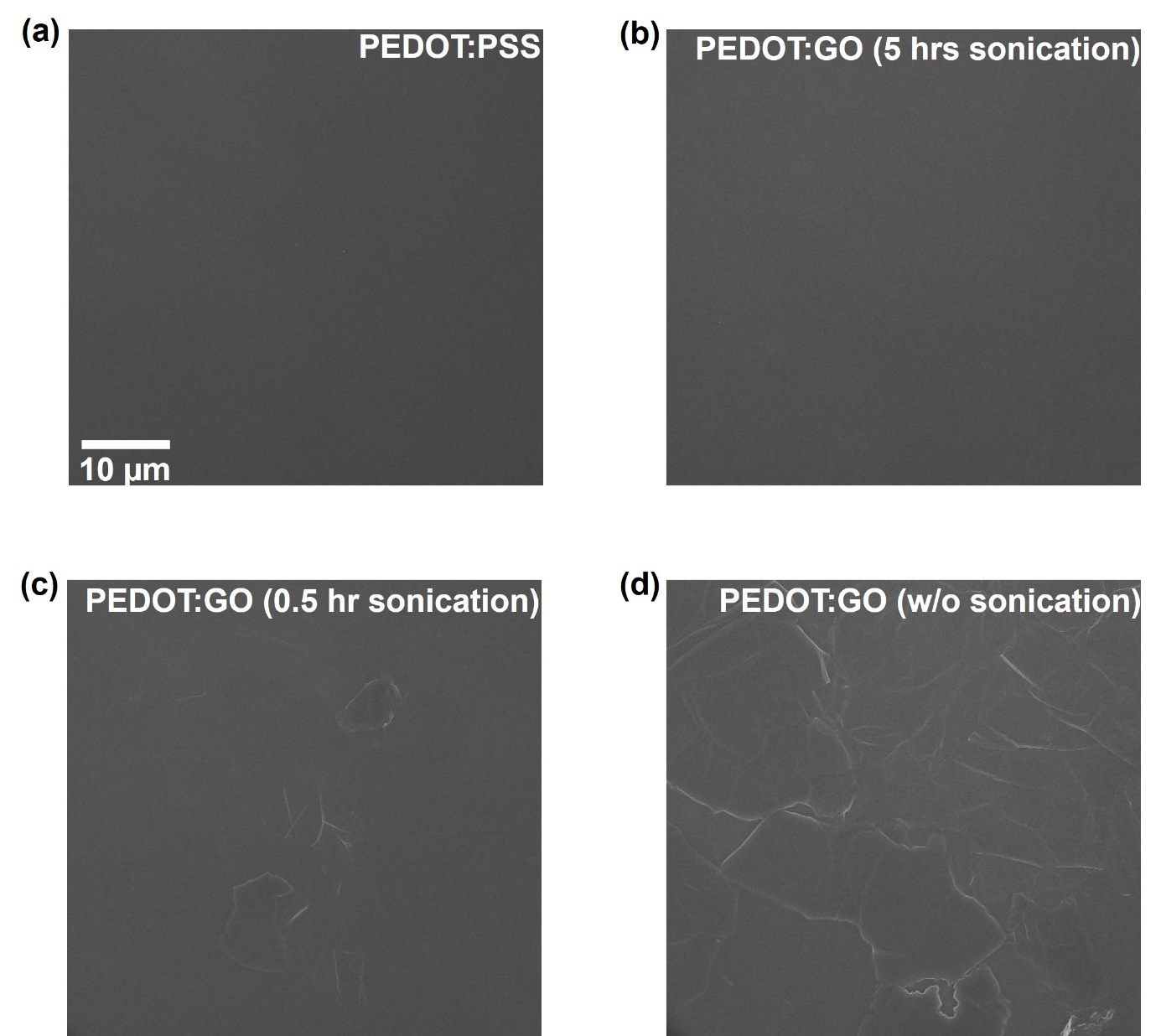
**

**Figure S2.** SEM top surface images of (a) PEDOT:PSS and (b-d) PEDOT:GO composite films with GO flakes prepared under various sonication times, respectively.


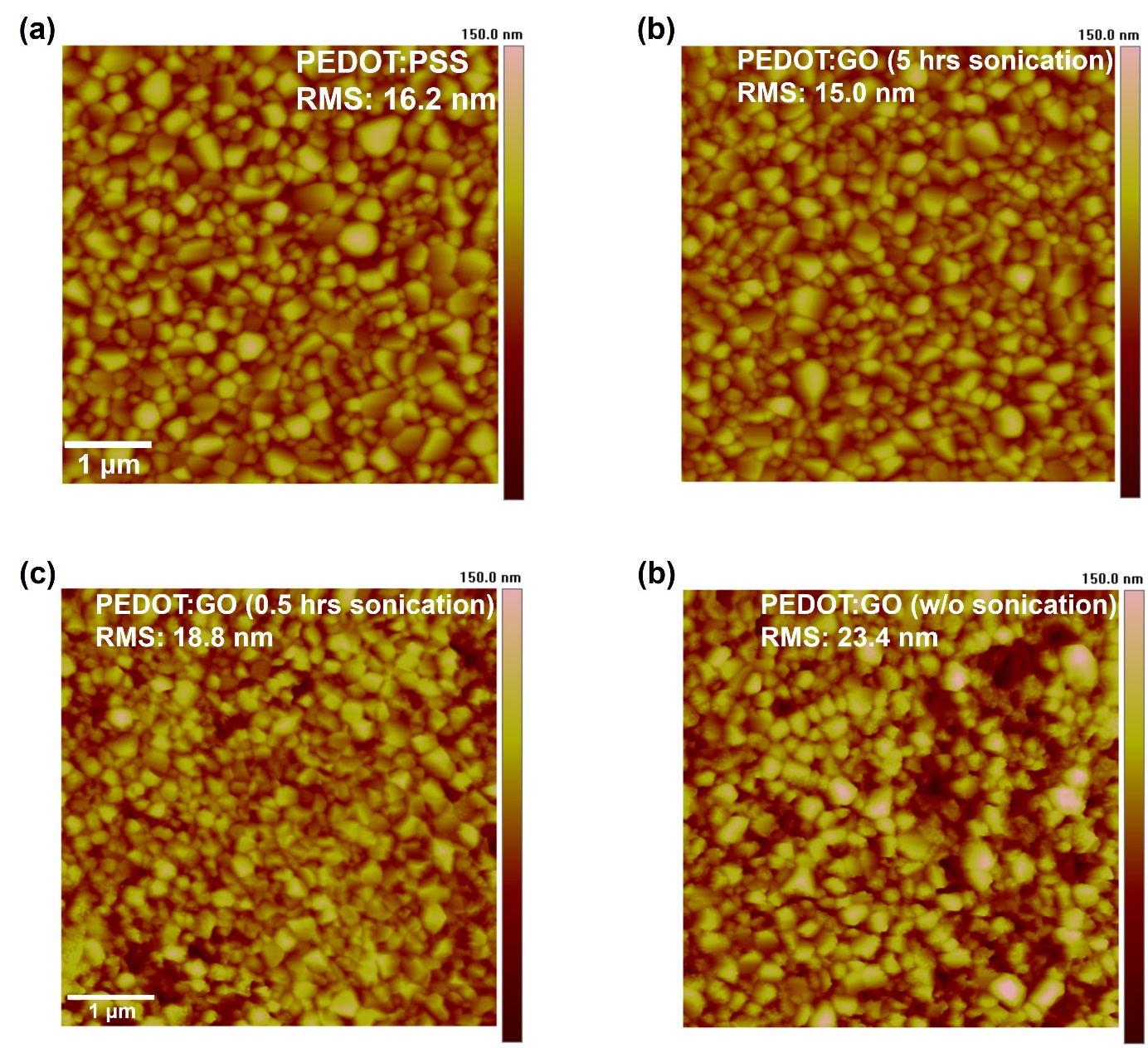


**Figure S3.** AFM images and RMS values of perovskite film with (a) PEDOT:PSS and (b-d) PEDOT:GO composite film prepared various sonication times.


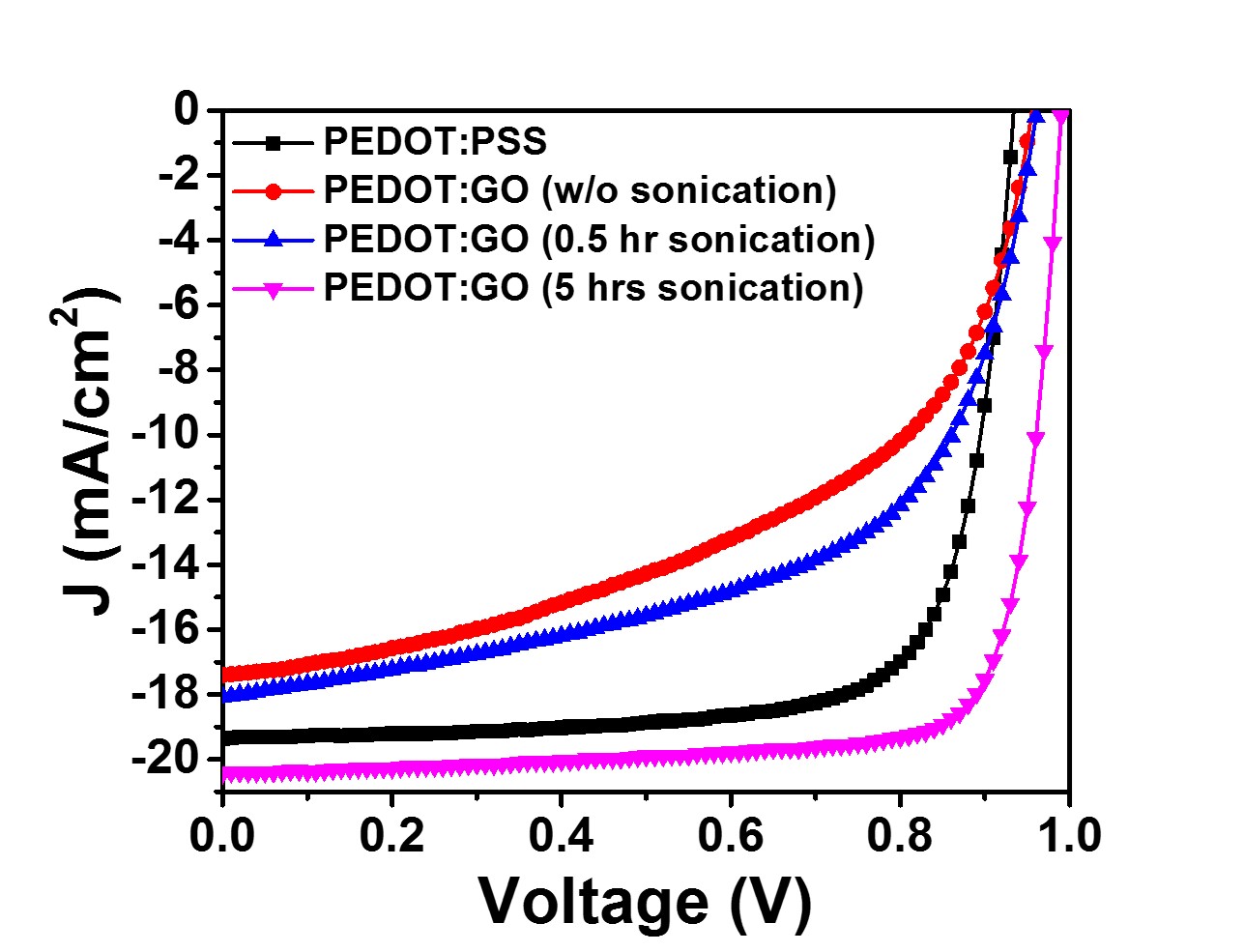


**Figure S4.** *J*-*V* curves measured under AM 1.5 simulated sunlight at 100 mW cm^-2^ equivalent irradiance for perovskite solar cells with PEDOT:PSS and PEDOT:GO composite layers (vol. ratio of 1:0.1) prepared under various sonication times.


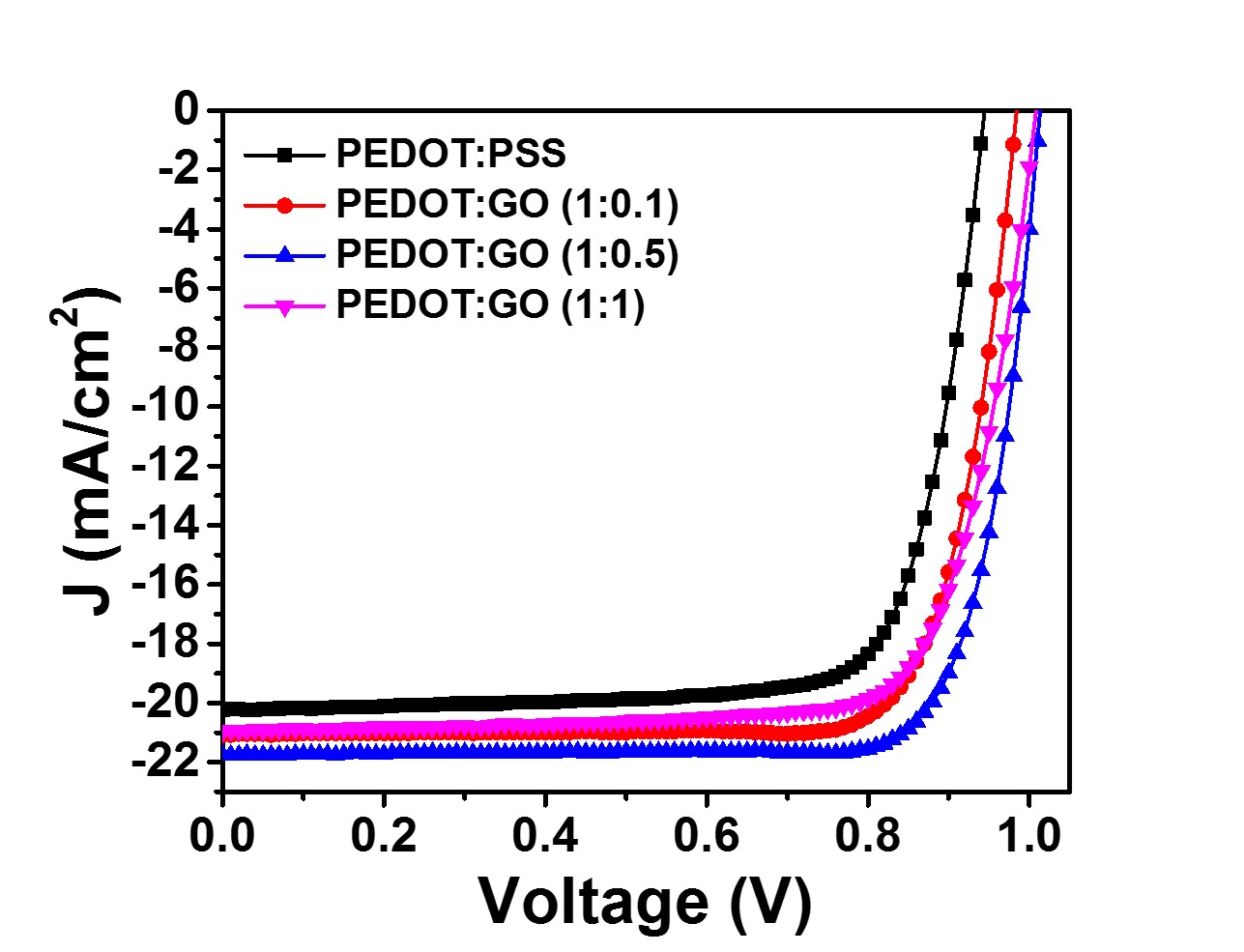


**Figure S5.** *J*-*V* curves measured under AM 1.5 simulated sunlight at 100 mW cm^-2^ equivalent irradiance for perovskite solar cells with various volume ratios of GO in PEDOT:GO film.

**
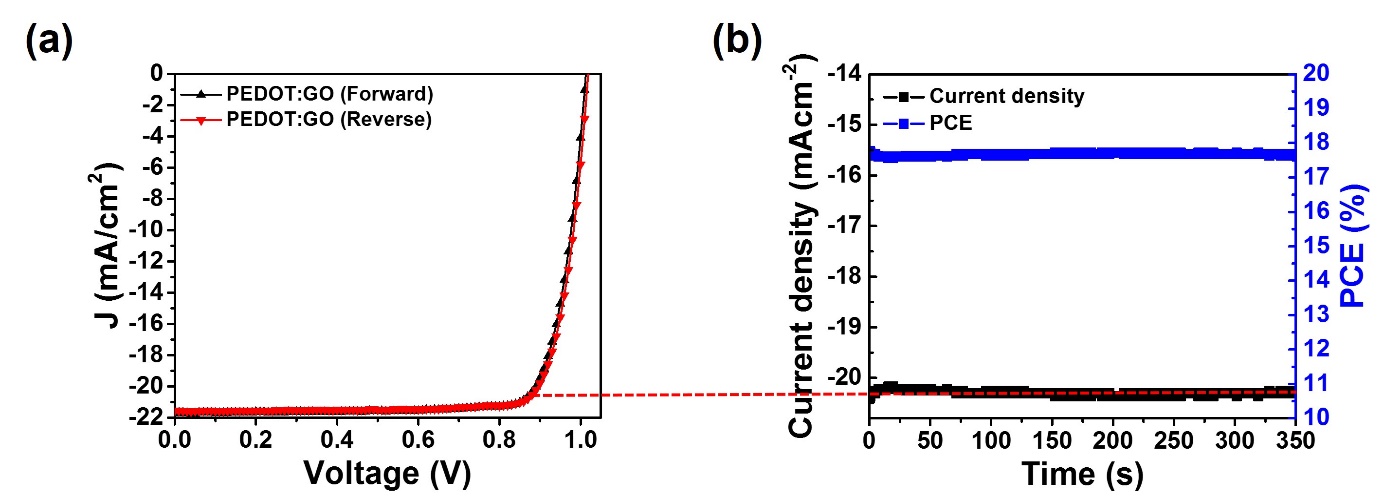
**

**Figure S6.** Photocurrent hysteresis and stabilized measurement for perovskite solar cell with PEDOT:GO composite film.


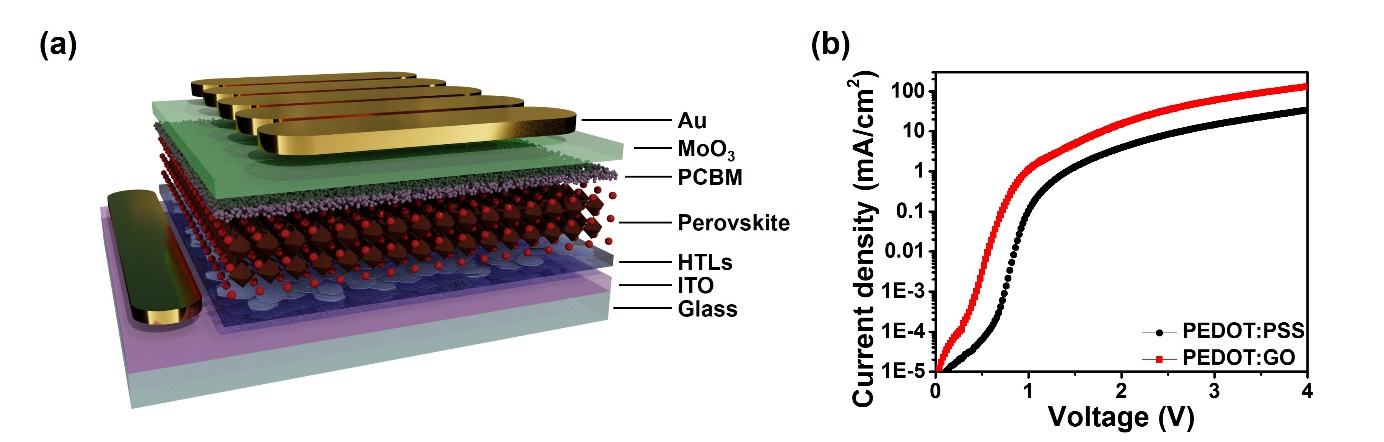


**Figure S7.** The device structure of the hole-only device (ITO/HTLs/perovskite/PCBM/MoO_3_/Au). (b) *J*-*V* characteristics of hole-only devices with PEDOT:PSS and PEDOT:GO composite layers.

**
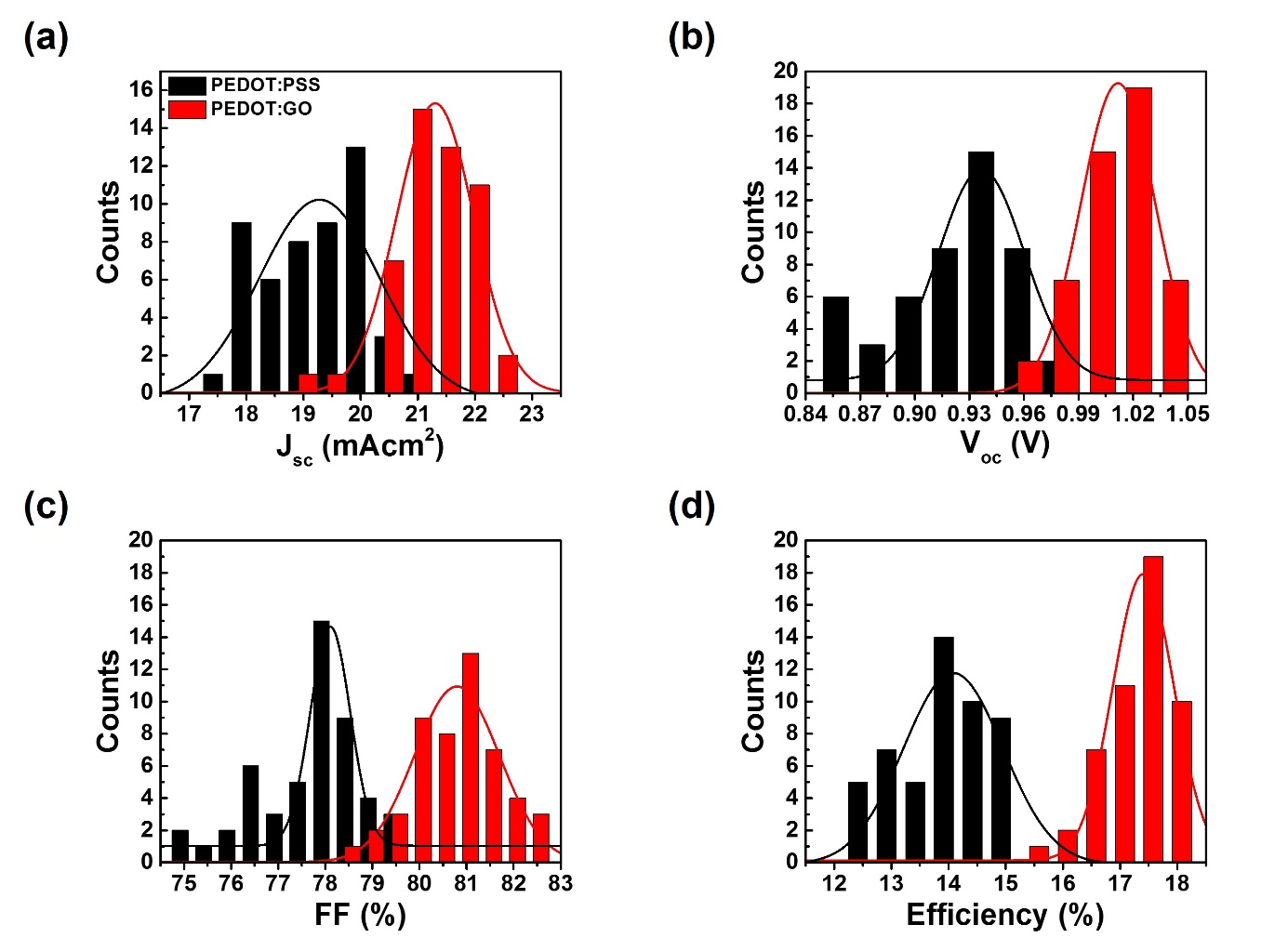
**

**Figure S8.** Histograms representing 50 samples each of PEDOT:PSS and PEDOT:GO composite layers in perovskite solar cells for (a) *J*_sc_ (b) *V*_oc_ (c) *FF* and (d) PCE.

**
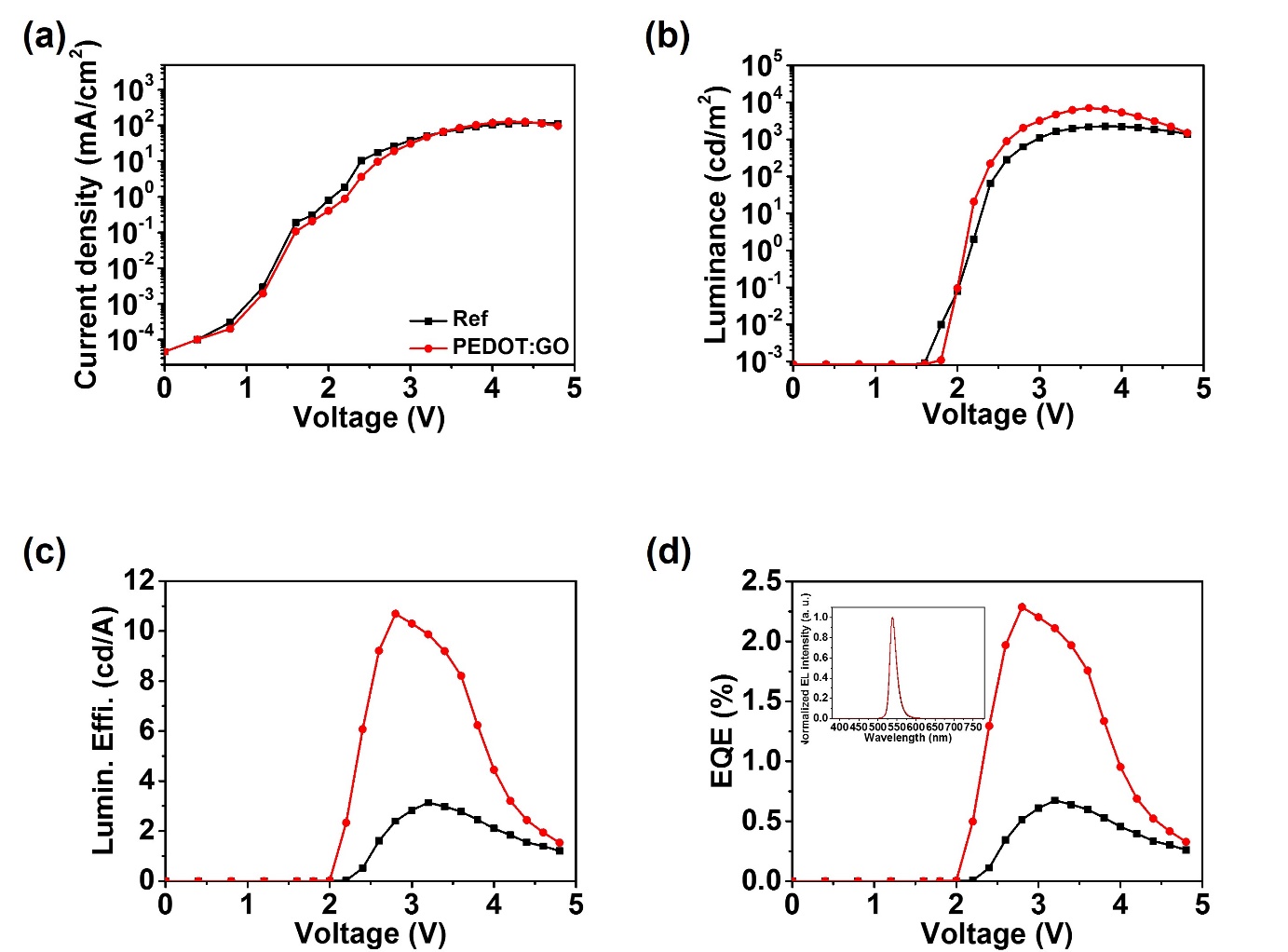
**

**Figure S9.** Light-emitting characterization of PeLEDs with PEDOT:PSS and PEDOT:GO composite layers in terms of (a) current density vs. voltage (*J*-*V*), (d) luminance vs. voltage (*L*-*V*), (c) luminous efficiency vs. voltage (*LE*-*V*), and (d) external quantum efficiency vs. voltage (*EQE*-*V*). The figure inset in (d) shows the EL spectra from PeLEDs with PEDOT:PSS and PEDOT:GO composite layers.

**Table S1. Summary of the device performance of perovskite solar cells with PEDOT:PSS and PEDOT:GO composite film prepared under different sonication times.**

| **Devices configuration** | **J _SC_ [mA/cm]** | **V _OC_ [V]** | **FF [%]** | **η[%]** |
| --- | --- | --- | --- | --- |
| **ITO/PEDOT:PSS/perovskite/PCBM/ZnO Nps/Ag** | **18.44±0.67** | **0.93±0.01** | **71.64±2.42** | **12.35±0.63** |
| **ITO/PEDOT:GO (w/o sonic.)**  **/perovskite/PCBM/ZnO Nps/Ag** | **16.82±0.75** | **0.96±0.01** | **46.85±2.92** | **7.55±0.73** |
| **ITO/PEDOT:GO (0.5 hr sonic.)**  **/perovskite/PCBM/ZnO Nps/Ag** | **17.33±0.73** | **0.97±0.01** | **53.67±2.45** | **8.98±0.59** |
| **ITO/PEDOT:GO (5 hrs sonic.)**  **/perovskite/PCBM/ZnO Nps/Ag** | **19.66±0.66** | **1.00±0.01** | **77.17±1.13** | **15.12±0.52** |

Average is based on 10 cells

**Table S2. Summary of the device performance of perovskite solar cells with various volume ratios of GO in PEDOT:GO solution.**

| **Devices configuration** | **J _SC_ [mA/cm]** | **V _OC_ [V]** | **FF [%]** | **η[%]** |
| --- | --- | --- | --- | --- |
| **ITO/PEDOT:PSS/perovskite/PCBM/ZnO NPs/Ag** | **19.20±0.55** | **0.94±0.01** | **73.18±1.70** | **13.21±0.62** |
| **ITO/PEDOT:GO (1:0.1)/**  **perovskite/PCBM/ZnO NPs/Ag** | **19.99±0.81** | **0.99±0.01** | **77.42±1.20** | **15.42±0.69** |
| **ITO/PEDOT:GO (1:0.5)/**  **perovskite/PCBM/ZnO NPs/Ag** | **20.99±0.64** | **1.01±0.01** | **78.70±1.11** | **16.69±0.71** |
| **ITO/PEDOT:GO (1:1)/**  **perovskite/PCBM/ZnO NPs/Ag** | **19.90±0.60** | **1.01±0.01** | **73.05±2.37** | **15.61±0.88** |

Average is based on 10 cells

**Table S3. Detailed exiction lifetime of perovskite, PEDOT:PSS/perovskite and PEDOT:GO/perovskite films with a PMMA as a encapsulation layer.**

| **sample** | ***τ*_1_(f_1_)(ns)** | ***τ*_2_(f_2_)(ns)** | ***χ*^2^** | ***τ*_avr_(ns)** |
| --- | --- | --- | --- | --- |
| **peorvskite/PMMA** | **106.65 (42.8)** | **33.12 (57.2)** | **1.151** | **64.17** |
| **PEDOT:PSS/peorvskite/PMMA** | **32.31 (31.0)** | **6.87 (69.0)** | **1.263** | **14.76** |
| **PEDOT:GO/perovskite/PMMA** | **6.48 (33.8)** | **2.50 (66.2)** | **1.241** | **3.48** |

**Table S4.** Fitting parameters of EIS plot of perovskite solar cell with PEDOT:PSS and PEDOT:GO composite films.

| **HTL** | ***R*_sheet_** | ***R*_internal_** | ***R*_surface_** |
| --- | --- | --- | --- |
| **PEDOT:PSS** | **12.66** | **357.8** | **60.80** |
| **PEDOT:GO** | **12.15** | **133.7** | **49.04** |

**Table S5. Summarized the conductivity of the PEDOT:PSS and PEDOT:GO compsite films on bare glass**

| **Sample** | **Conductivity (S/cm)** |
| --- | --- |
| **Glass/PEDOT:PSS** | **0.011** |
| **Glass/PEDOT:GO** | **0.048** |

**Table S6. Summary of the device performances of PeLEDs with PEDOT:PSS and PEDOT:GO composite layer.**

| **Devices configuration** | **L _max_ [cd/m^2^]**  **@ bias** | **LE _max_ [cd/A]**  **@ bias** | **EQE _max_ [%]**  **@ bias** |
| --- | --- | --- | --- |
| **ITO/PEDOT:PSS/perovskite/TPBi/LiF/Ag** | **2,274 @ 3.8** | **3.14 @ 3.2** | **0.67 @ 3.2** |
| **ITO/PEDOT:GO/perovskite/TPBi/LiF/Ag** | **7,047 @ 3.6** | **10.70 @ 2.8** | **2.29 @ 2.8** |
